# Mouse innate-like B-1 lymphocytes promote inhaled particle-induced in vitro granuloma formation and inflammation in conjunction with macrophages

**DOI:** 10.1007/s00204-021-03200-2

**Published:** 2021-12-21

**Authors:** Léa Hiéronimus, Raïssa Demazy, Laura Christiaens, Francine Uwambayinema, Jean-François Geuens, Youssof Yacoub, François Huaux

**Affiliations:** Louvain Centre for Toxicology and Applied Pharmacology (LTAP), Institut de Recherche Expérimentale et Clinique (IREC), Université Catholique de Louvain (UCLouvain), Avenue Hippocrate 57, bte B-1.57.06, 1200 Brussels, Belgium

**Keywords:** B-1 lymphocytes, Macrophages, Innate immunity, Granuloma, Inflammation, Silica, Nanoparticles

## Abstract

The current paradigm for explaining lung granulomatous diseases induced by inhaled particles is mainly based on macrophages. This mechanism is now challenging because B lymphocytes also infiltrate injured tissue, and the deficiency in B lymphocytes is associated with limited lung granulomas in silica-treated mice. Here, we investigated how B lymphocytes respond to micro- and nanoparticles by combining in vivo and in vitro mouse models. We first demonstrated that innate-like B-1 lymphocytes (not conventional B-2 lymphocytes or plasma cells) specifically accumulated during granuloma formation in mice instilled with crystalline silica (DQ12, 2.5 mg/mouse) and carbon nanotubes (CNT Mitsui, 0.2 mg/mouse). In comparison to macrophages, peritoneal B-1 lymphocytes purified from naïve mice were resistant to the pyroptotic activity of reactive particles (up to 1 mg/mL) but clustered to establish in vitro cell/particle aggregates. Mouse B-1 lymphocytes (not B-2 lymphocytes) in coculture with macrophages and CNT (0.1 µg/mL) organized three-dimensional spheroid structures in Matrigel and stimulated the release of TIMP-1. Furthermore, purified B-1 lymphocytes are sensitive to nanosilica toxicity through radical generation in culture. Nanosilica-exposed B-1 lymphocytes released proinflammatory cytokines and alarmins. In conclusion, our data indicate that in addition to macrophages, B-1 lymphocytes participate in micrometric particle-induced granuloma formation and display inflammatory functions in response to nanoparticles.

**Supplementary Information:**

The online version contains supplementary material available at 10.1007/s00204-021-03200-2.

## Introduction

Excessive inhalation of toxic particles has been associated with the development of multiple respiratory manifestations, such as granuloma formation, fibrosis and cancer (Bierkandt et al. 2018). The accumulation of particles in the pulmonary alveoli causes tissue damage and activates resident macrophages. During this acute response to particles, these phagocytes execute essential functions by clearing particles. Reactive microsized materials such as silica and carbon nanotubes (CNT) disrupt phagocytosis and induce pyroptosis, limiting tissue translocation, particle biodegradation and clearance. These biopersistent particles constantly activate resident macrophages and polarize them in an inflammatory-like M1 state, leading to necrosis and the alarmin release cycle. Activated M1 macrophages also promote the release of chemokines and cytokines by epithelial and endothelial cells, the concentration gradient of which attracts various immune cells comprising additional recruited macrophages and neutrophils as well as T and B lymphocytes (Laskin et al. 2019; Malaviya et al. 2020).

The resolution of lung inflammation induced by particles is characterized by the switch from inflammatory M1-polarized macrophage enrichment to more anti-inflammatory/immunosuppressive (M2) polarization (Lim et al. 2020; Xiang et al. 2016). In response to various foreign bodies, including particles, some M2 macrophages aggregate into compact granulomas to prevent the dispersion of foreign bodies and their toxic products toward healthy tissues (Trout and Holian 2020b), while others fuse to form plurinuclear giant cells, which increases their phagocyte capacity but impairs their cytokine release/production (Brooks et al. 2019; Milde et al. 2015). The current theory is that foreign body-associated plurinucleated giant cell formation serves the same function as granulomas at a smaller scale, that is, to isolate the host from the foreign body to prevent excessive inflammation (Trout and Holian 2020a). During the chronic phase, granuloma M2-polarized macrophages participate in the development of fibrosis by activating fibroblasts and induce exaggerated tissue repair (Lebrun et al. 2017; Zhao et al. 2020). The persistence of this process leads to excessive extracellular matrix protein deposition and irreversible granulomatous fibrous scarring, thereby decreasing lung capacity (Malaviya et al. 2020). Preventing abnormal extracellular matrix remodeling after reactive particle instillation using metalloproteinase inhibitor-deficient mice markedly reduces fibrotic granulomatous lesion progression (Mohan et al. 2020).

The size of the particle represents a key parameter for interpreting lung toxicity. Nanoparticles present an enhanced ability to penetrate intracellular targets in the lung and systemic circulation. It is now accepted that nanoparticles are more cytotoxic and cause more severe acute inflammatory responses than microparticles. Because of their high penetrative property, nanoparticles are cleared out of the organism rapidly, resulting in fewer fibrotic lesions (Napierska et al. 2010). However, certain studies show that the inhalation of nanoparticles can also cause pulmonary granulomas in the late stages, as nanoparticle aggregates can induce responses attributed to microparticles (Zhu et al. 2016).

While the role of macrophages in granuloma formation and subsequent fibrosis is undisputed, additional immune cells were unexpectedly involved in the pathogenic process. Indeed, it has been observed that B lymphocyte-deficient mice treated with inorganic (silica, beryllium and pristine oil) (Arras et al. 2006; Atif et al. 2019; Chen et al. 2010) or organic (bacteria, fungi and parasite) (Ji et al. 2008; Russo and Mariano 2010; Tristão et al. 2013) foreign bodies developed fewer lung fibrotic granulomas than wild-type animals.

B lymphocytes are composed of several subsets that comprise conventional-like B-2 lymphocytes, regulatory B lymphocytes (Bregs, including interleukin-10-producing B10 cells), plasma cells, marginal zone (MZ) B lymphocytes and innate-like B-1 lymphocytes. B-2 lymphocytes and plasma cells are responsible for adaptive immune responses and generate high-affinity antibodies with precise antigen specificity. In contrast, B-1 and MZ B lymphocytes produce low-affinity antibodies with broad reactivity. B-1 lymphocytes maintain natural IgM and IgA levels, acquire immune stimulatory or regulatory activities by releasing pro- or anti-inflammatory cytokines and play critical roles in autoimmunity, inflammation and infection (Berland and Wortis 2002; Yoshimoto 2020). They also interact with fibroblasts through fibroblast-related factors such as TNF-α, IL-10, IL-6 and prostaglandin E2 (PGE2) (Arcanjo et al. 2017; Arras et al. 2006; Barbosa et al. 2018).

Interestingly, in addition to antibody production, B-1 lymphocytes share many characteristics with macrophages with which they crosstalk. Both cell types are able to express myeloid cell markers such as F4/80, CD11b, MHC II, and CD80/CD86 and can act as phagocytes, present antigens and stimulate T cells (Ghosn et al. 2006; Popi et al. 2012). Peritoneal macrophages ensure B-1 lymphocyte proliferation (Almeida et al. 2001; Thies et al. 2013), homing in the peritoneum (Ansel et al. 2002; Ito et al. 2004) and migration in the intestines to produce IgA antibodies (Okabe and Medzhitov 2014). Reverse collaboration is also possible; by secreting cytokines such as MCP1 (CCL2), GM-CSF and IL-10, B-1 lymphocytes modify macrophage function and polarization (Ahmed and Koma 2015; Chin et al. 2019).

This study determines the contribution of innate-like B-1 lymphocytes in response to reactive particles. We used mouse lung and peritoneal cells to delineate their recruitment and function in response to micrometric and nanometric particles compared to or in association with macrophages. Our results provide a crucial role of B-1 lymphocytes in granuloma formation and inflammatory responses. We show that B-1 lymphocytes form micrometric cell/particle clusters and potentiate in vitro granuloma-like structure formation and extracellular matrix remodeling in combination with M2 macrophages. They are also able to sense nanoparticles and release master proinflammatory cytokines.

## Materials and methods

### Particles

Particles used in this study were crystalline silica DQ12 (SiO_2_, d50 = 2.2 µm; DMT, Essen, Germany); crystalline Min-U-Sil^®^ 5 (d50 = 1.6 µm, US Silica Company, Berkeley Springs, West Virginia, USA); monodisperse silica spheres (MSS, d50 = 1 µm, Fiber Optic Center Inc., New Bedford MA, USA); and nanosized silica (Aerosil OX50 and FK 320, respectively 40 nm and 15 nm, Evonik, Degussa, Frankfurt AM, Germany), carbon black (CB, ultrafine, d50 = 35 nm, ENSACO 250G, Timcal), carbon nanotubes (CNT/CNT-7/MWCNT-7, *d* = 75 nm, *L* = 7.1 µm, Mitsui, Tokyo, Japan), asbestos (crocidolite, *d* = 200 nm, *L* = 3 µm, UICC, Geneva, Switzerland), tungsten carbide (WC, d50 < 1 µm; Johnson Matthey, Royston, United Kingdom) and cobalt oxide (Co3O4, d50 < 10 μm, Sigma Aldrich, St. Louis, Missouri, USA, now part of Merck). To sterilize and inactivate any trace of endotoxin, particles were heated at 200 °C for 2 h.

### Animals

Female C57BL/6 mice aged 2–3 months were provided by Janvier SAS (St. Berthevin, France). Suspensions of SiO_2_ (2.5 mg/mouse), WC (2.5 mg/mouse) and CNT (0.2 mg/mouse) were directly injected into the lung by oropharyngeal aspiration. The control mice received 50 μL of NaCl. The experimental protocol complied with Belgian and European regulations (EEC n ° 86/609, LA1230312 and 2018/UCL/MD/012).

### Tissue sample and cell suspension collection

A digestion solution containing 100 µL of pronase 20 mg/mL (Protease type XIV, from *Streptomyces griseus*, Sigma-Aldrich) and 100 µL of DNAse 1 mg/mL (deoxyribonuclease I type IV from bovine pancreas, Sigma-Aldrich) in 800 µL of HBSS solution at 37 °C was injected into the lungs and incubated for 20 min. Tissues were then harvested and mechanically dissociated to obtain lung cell suspensions (Huaux et al. 2018). The perithymic lymph nodes and spleen were washed in DPBS and HBSS and then crushed to release cells. Femoral bone marrow was recovered by the injection of HBSS through the bone ends. Cell suspensions were passed through a 70-µm filter, and red blood cells were lysed (RBC lysis buffer, dilution 10×, eBiosciences, Thermo Fisher Scientific, Waltham, Massachusetts, USA). Peritoneal lavage was collected by injecting 10 mL of NaCl into the peritoneal cavity.

### Cell purification, culture and recovering

For early cytotoxicity and cytokine release, B-1 cells were obtained from peritoneal CD19^+^ B lymphocytes isolated using anti-CD19 antibodies coupled with magnetic beads (anti-CD19 microBeads, mouse, Miltenyi Biotec, Bergisch Gladbach, Germany) and the company protocol. CD19^−^ adherent cells corresponded to macrophages. The separate fractions were incubated for 24 h in RPMI (for CD19^+^ B lymphocytes) or DMEM (for adherent CD19^−^ macrophages) containing 10% FBS (fetal bovine serum, Thermo Fisher Scientific) in a humidified incubator at 37 °C with 5% CO_2_. In a 96-well plate (Flat bottom, CellStar, Greiner, Sigma-Aldrich), 150,000–200,000 cells per well were incubated in serum-free RPMI culture medium and exposed to particles for 24 h. For granuloma-like structure and granuloma formation, peritoneal B-1 cells were expanded in vitro by a method described elsewhere (Almeida et al. 2001). M2 macrophages were differentiated from bone marrow progenitors in DMEM cell medium (Dulbecco’s modified Eagle’s medium from Gibco, Thermo Fisher Scientific) + 10% decomplemented FBS (+ 1% antibiotic–antimycotic from Thermo Fisher Scientific) + 20 ng/mL recombinant mouse M-CSF (R&D Systems, Bio-Techne, Minneapolis, Minnesota, USA) for 6 days (Rios et al. 2017; Sanchez et al. 2011). Spleen CD19^+^ cells were used as B-2 lymphocytes (Arras et al. 2006). B-1 lymphocytes, macrophages and B-2 lymphocytes were counted, and 50,000 B lymphocytes and/or macrophages were incubated in RPMI culture medium with 10% decomplemented FBS and 1% antibiotic–antimycotic for 6 days in wells coated with Matrigel (Corning, Thermo Fisher Scientific) according to the 3D-cell culture technique used for granuloma formation by Cronan et al. (2018).

To inhibit interactions of class A scavenger receptors with particles, fucoidan (Fucus Vesiculosus F8190, Sigma-Aldrich) or dextran sulfate sodium salt (*Leuconostoc* spp. D8906, Sigma-Aldrich) was added to the cells 1 h before exposure to the particles (Bonilla et al. 2013). Phagocytosis was blocked 2 h before particle exposure by adding cytochalasin D (Zygosporium mansonii, 5 mg/mL in DMSO, 0.2 µm filtered C2618, Sigma-Aldrich) at 2.5 µg/mL or incubating cells at 4 °C for 2 h (Bonilla et al. 2013). To inhibit free radical formation, catalase (from bovine Liver C1345 Sigma-Aldrich) was added to the particles (2000 U/mL) just before exposure (Bechtel and Bauer 2009).

### FACS and microscopy

Cells were incubated with Fc block (anti-CD16 and anti-CD32; BD Biosciences, Franklin Lakes, New Jersey, USA) and different fluorochrome-coupled antibodies as follows: anti-CD45 (clone 30-F11; FITC; BD Biosciences), anti-CD5 (clone 53-7.3; PE and PE-Vio770; Miltenyi Biotec), anti-CD11b (clone REA592, Vioblue; Miltenyi Biotec), anti-CD22 (clone Cy34.1; Pe-Vio770; Miltenyi Biotec), anti-CD23 (clone B3B4; APC; Miltenyi Biotec), anti-CD38 (clone 90.4; APC; Miltenyi Biotec), anti-CD43 (clone REA840; PE; Miltenyi Biotec) and anti-CD138 (clone REA104; PE; Miltenyi Biotec), anti-CD19 antibody (clone RE749; APC-770; Miltenyi Biotec). The analysis was performed by FACS CANTO II (BD Biosciences) and FlowJo V10 software. Positive populations were identified using fluorescence minus one (FMO) controls. The culture wells were analyzed with a Leitz labovert microscope equipped with an axiocam MRc camera (Zeiss, Zaventem, Belgium). For all pictures of each well, compact cell aggregates were delimited, and their areas were measured using the Axiovision program. The cell aggregates as presented in the graphs were obtained by the sum of all measured areas per well.

### Relative cell activity, alarmins, cytokines and metalloproteinase inhibitors

The cellular ATP content was measured using the CellTiter-Glo^®^ Luminescent Cell Viability Assay (Promega, Madison, Wisconsin, USA) according to the manufacturer’s instructions. Lactate dehydrogenase (LDH) was measured according to the LDH cytotoxicity assay kit protocol (Cayman Chemical, Uden, Netherlands). The supernatants of peritoneal macrophages and B-1 lymphocytes were collected 24 h after particle treatments for cytokine measurement. IL-1α, IL-1β and tumor necrosis factor (TNF)-α were quantified by enzyme-linked immunosorbent assay (ELISA) (DuoSet ELISA, R&D Systems) following the manufacturer’s instructions. For IL-1β quantification, cells were stimulated with 0.1 µg/mL LPS (LPS Serotype EH100, ENZO Life Sciences, now part of Thermo Fisher Scientific) 2 h before particle exposure as a positive control. The supernatant of M2 macrophages with or without B cells was collected for tissue inhibitor of metalloproteinases-1 (TIMP-1) measurement by ELISA (DuoSet ELISA, R&D Systems).

### Statistics

Unless specified in the legend, the results were analyzed via ANOVA followed by a Dunnett test. The significance threshold, alpha, was fixed at 0.05. *p < 0.05, **p < 0.01 and ***p < 0.001 indicate significant differences compared to the control. B-Lymphocyte populations were analyzed simultaneously in all treated mice at the same time point. As statistical analyses (one-way ANOVA and Dunnett’s test) revealed no significant change in control mice, their results were combined in the graphs, labelled as “CTRL”. In vitro analysis was carried out up to three times with three replicates. Graphs and analyses were performed using GraphPad Prism Software. Bars represent means ± SD. A graphical abstract cartoon was generated using Biorender.

## Results

### B-1 lymphocytes specifically accumulate during the development of particle-induced pulmonary granuloma and fibrosis

**Fig. 1:**
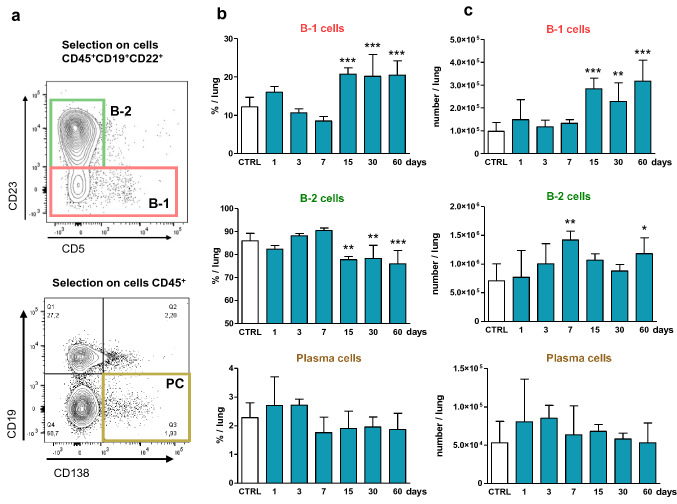
Late and specific accumulation of lung B-1 lymphocytes after silica treatment. **a** Gating strategy in flow cytometry of B-2 lymphocytes (CD23^+^) and B-1 lymphocytes (CD23^−^) from CD45^+^ CD19^+^ CD22^+^ cells or plasma cells (CD45^+^ CD19^−^ CD138^+^) in the lung; representative images of the populations obtained from mouse lungs 15 days after instillation of NaCl. **b**, **c** Proportions (**b**) and numbers (**c**) of B-1 lymphocytes, B-2 lymphocytes and plasma cells in the lungs of control mice and of mice treated with silica (1, 3, 7, 15, 30 and 60 days after instillation of SiO_2_, *n* = 5 per time, 2.5 mg/mouse). The control column (CTRL) shows the combined results obtained from control mice analyzed at each studied time point (*n* = 14, no significant difference between control groups, see “Material and methods”)

**Fig. 2:**
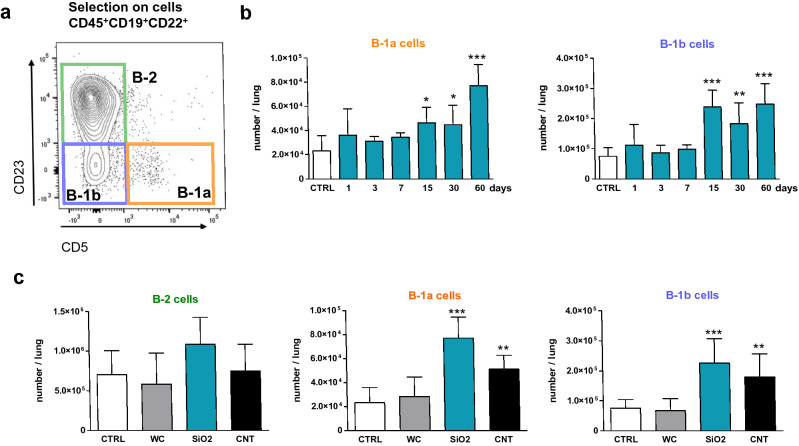
B-1a and B-1b subpopulations accumulate in silica-treated lungs during fibrotic granuloma formation. **a** Gating strategy in flow cytometry of B-2 lymphocytes (CD23^+^), B-1a cells (CD23^−^ CD5^+^) and B-1b lymphocytes (CD23^−^ CD5^−^) from CD45^+^ CD19^+^ CD22^+^ cells; representative images of the populations obtained from mouse lungs 15 days after instillation of NaCl. **b** Numbers of B-1a and B-1b lymphocytes in the lungs of control mice and of mice treated with silica (1, 3, 7, 15, 30 and 60 days after instillation of SiO_2_, *n* = 5 per time, 2.5 mg/mouse). The control column (CTRL) shows the combined results obtained from control mice analyzed at each studied time point (*n* = 14, no significant difference between control groups, see “Material and methods”). **c** Numbers of pulmonary B-2, B-1a and B-1b lymphocytes in the control group (CTRL) or mice treated with tungsten carbide (WC, *n* = 3, 2.5 mg/mouse), silica (SiO_2_, *n* = 5, 2.5 mg/mouse) or carbon nanotubes (CNT, *n* = 4, 0.2 mg/mouse) 60 days after instillation

We first determined which B lymphocyte subpopulations accumulate in pulmonary tissue during silica-induced short- and long-term responses (1–7 days and 15–60 days, respectively) in C57BL/6 mice compared to saline-treated mice. The proportion and number of B-1 (CD45^+^ CD19^+^ CD22^+^ CD23^−^) and B-2 (conventional B lymphocytes, CD45^+^ CD19^+^ CD22^+^ CD23^+^) lymphocytes and plasma cells (CD45^+^ CD19^−^ CD138^+^) were examined after lung tissue dissociation (Fig. 1a, see also Supplementary file, Fig. S1a for the gating strategy). Control lungs contain a majority of B-2 lymphocytes (86%) compared to B-1 lymphocytes (12%) and plasma cells (2%). A significant and constant increase in the percentage of pulmonary B-1 lymphocytes was observed at late time points after silica treatment (± 20% from day 15 to day 60, Fig. 1b) but not at early points (1–7 days). The proportion of B-2 lymphocytes decreased while plasma cells remained constant. The total numbers of lung B-1 lymphocytes were also increased during the long-term responses to silica, while B-2 lymphocytes and plasma cell numbers remained unmodified (Fig. 1c). We additionally analyzed B-1a (CD5^+^) and B-1b (CD5^−^) lymphocytes (Fig. 2a) and noted that the numbers of these two B-1 subpopulations were significantly increased from day 15 to day 60 (Fig. 2b). These data indicate that B-1 (a/b) lymphocytes specifically arise in the lungs during the chronic stage of fibrotic granuloma development.

We then determined whether the accumulation of B-1 lymphocytes is specific to granulomagenic and fibrotic particles. Animals were then treated with tungsten carbide (WC), an inert particle that does not induce granuloma or fibrosis (Huaux et al. 1999), and carbon nanotubes (CNT), which induce robust fibrotic granulomas (Khaliullin et al. 2017). As silica, CNT induced lung B-1 a/b lymphocyte accumulation 60 days after treatment, while WC did not impact B-1 lymphocyte numbers in comparison to controls (Fig. 2c). Lung B-2 lymphocyte numbers were not modified by particle treatments.

Finally, we determined whether the pulmonary accumulation of B-1 lymphocytes after silica was systemic. The number of B-1a and B-1b lymphocytes in lung-associated lymph nodes (LALN) progressively increased and became significant 60 days after treatment (Supplementary file, Fig. S2a). Analysis of the spleen revealed the accumulation of B-1b lymphocytes during long-term responses to silica (15 and 60 days) but not B-1a lymphocytes. Similar results were observed after CNT instillation but not after WC treatment (not shown). Our data show that the local effect of silica on B-1 lymphocyte populations is extended to the spleen and lymph nodes.

### B-1 lymphocytes are functionally distinct from M2-like and M1-like macrophages

M2 immunosuppressive macrophages infiltrate the lungs of mice during the development of particle-induced fibrotic granulomas and activate fibroblasts through the release of anti-inflammatory mediators (Malaviya et al. 2020). The specific accumulation of B-1 lymphocytes in the lung during the same period and their shared macrophage functions suggested that these B lymphocytes may play a similar pathogenic role to M2 macrophages. We demonstrated, however, that recruited B-1 lymphocytes do not correspond to M2 macrophages because purified lung B-1 lymphocytes from silica-treated mice did not activate fibroblasts, produce IL-10 or inhibit T cell proliferation (Supplementary file, Fig. S3a–d). These data suggest that recruited B-1 lymphocytes possess distinct pathogenic function(s) from M2 macrophages.

**Fig. 3:**
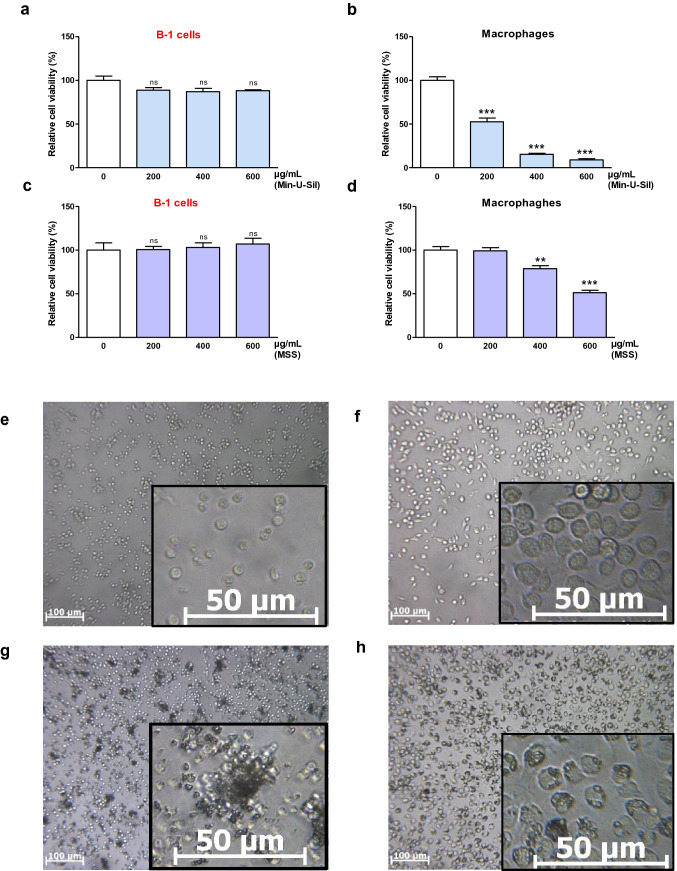
B-1 lymphocytes respond differently to micrometric particles than macrophages and cluster instead of phagocytose micrometric particles. **a**–**d** Relative cellular activity (ATP) measured in peritoneal B-1 lymphocytes (**a**, **c**) and macrophages (**b**, **d**) 24 h after exposure to different doses of micrometric silica particles (Min-U-Sil **a**, **b** and MSS **c**, **d**) compared to unexposed cells. **e**–**h** Representative optical microscopy images of peritoneal B-1 lymphocytes (**e**, **g**) and macrophages (**f**, **h**) 24 h after exposure to medium (**e**, **f**) or to micrometric silica (DQ12, 40 µg/mL) (**g**, **h**)

We verified whether B-1 lymphocytes possess comparable early inflammatory capability to M1 macrophages when exposed to micrometric silica (pyroptosis and IL-1β release). Purified peritoneal B-1 lymphocytes and macrophages from naïve mice were exposed for 24 h to a range of microsized silica doses. In contrast to macrophages, B-1 lymphocytes were resistant to the pyroptotic activity of crystalline Min-U-Sil and amorphous MSS silica (estimated by cell survival assessment) when exposed to the same doses (Fig. 3a–d). We additionally showed that B-1 lymphocytes did not release pyroptotic IL-1β after silica exposure, in contrast to macrophages (Supplementary file, Fig. S3e). Our data thus indicate that B-1 lymphocytes have no proximity to M1 macrophages and possess other functions when exposed to micrometric and granulomagenic particles.

### B-1 lymphocytes cluster micrometric particles and promote in vitro granuloma-like structure formation in the presence of M2 macrophages

**Fig. 4:**
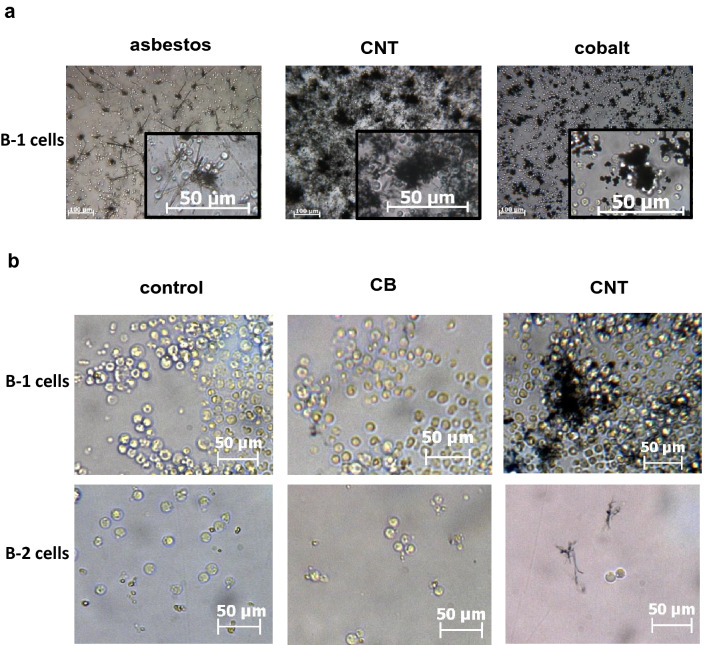
Particle clustering is specific to the B-1 lymphocyte subset and dependent on the granulomagenic activity of micrometric particles. **a** Representative optical microscopy images of peritoneal B-1 lymphocytes exposed to 100 μg/mL micrometric and granulomagenic crocidolite, CNT or cobalt oxide (Co_3_O_4_) at 24 h. **b** Representative optical microscopy images of peritoneal B-1 lymphocytes and spleen B-2 lymphocytes after 6 days unexposed or exposed to 0.1 µg/mL inert carbon black (CB) or to 1 µg/mL carbon nanotubes (CNT) for 6 days

Interestingly, our in vitro experiment revealed the marked micrometric particle-handling capacity of B-1 lymphocytes since they were mainly clustered around particles while macrophages phagocytosed particles (Fig. 3e–h). We then confirmed these observations by exposing purified B-1 lymphocytes to other granulomagenic particles and showed that CNT, crocidolite, and cobalt oxide (Cho et al. 2012; Sanchez et al. 2011) also induced cell/particle aggregates (Fig. 4a, b). Particle clustering by B-1 lymphocytes was not observed with nongranulomagenic carbon black (CB) (Fig. 4b). Particle clustering was B-1 lymphocyte-specific, as particles were not grouped together with B-2 lymphocytes (Fig. 4b). Altogether, these results indicate that B-1 lymphocytes specifically respond to microsized and granulomagenic particles by forming cell/particle clusters.

**Fig. 5:**
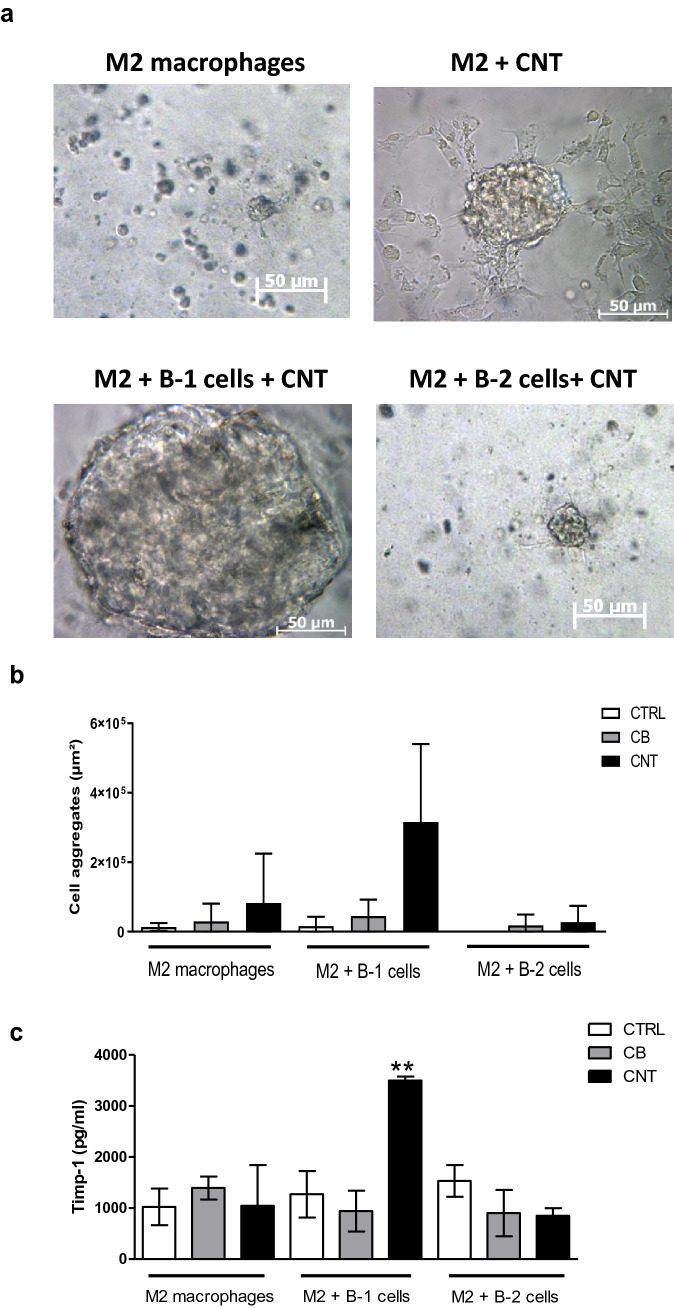
B-1 lymphocytes promote in vitro granuloma formation in the presence of M2 macrophages. **a**–**c** Representative optical microscopy images illustrating cell aggregates (**a**), surface occupied in the culture wells by cell aggregates (**b**) and TIMP-1 levels in the supernatant (**c**) of M2 macrophages with or without B-1/B-2 cells, when unexposed (CTRL), exposed to 1 µg/mL carbon black (CB) or exposed to 0.1 µg/mL carbon nanotubes (CNT) in culture for 6 days in Matrigel

From their clustering capacity, we hypothesized that B-1 lymphocytes could collaborate with macrophages in particle-induced granuloma formation and investigated their possible collaboration in organizing granuloma-like structures in vitro. Adding peritoneal B-1 lymphocytes to bone marrow-derived M2 macrophages increased the size of cell aggregates when these cells were exposed to CNT but not to CB (Fig. 5a, b). These well-defined three-dimensional structures in Matrigel were not observed by combining M2 macrophages and B-2 lymphocytes or in macrophages seeded alone and exposed or not to CNT. These results indicate that B-1 lymphocytes promote the formation of granuloma-like structures in conjunction with M2 macrophages.

Then, we investigated the possible mediator(s) by which B-1 lymphocytes promote the formation of granuloma-like structures and found that adding B-1 (and not B-2) lymphocytes to M2 macrophages further increased the levels of tissue inhibitor metalloproteinase 1 (TIMP-1) when exposed to granuloma-inducing CNT (Fig. 5c). Taken together, these results indicate that B-1 lymphocytes specifically respond to microsized granulomagenic particles and organize three-dimensional granuloma-like structures in conjunction with macrophages by increasing matrix protein accumulation.

### B-1 lymphocytes are sensitive to nanoparticles in an ROS-dependent manner

**Fig. 6:**
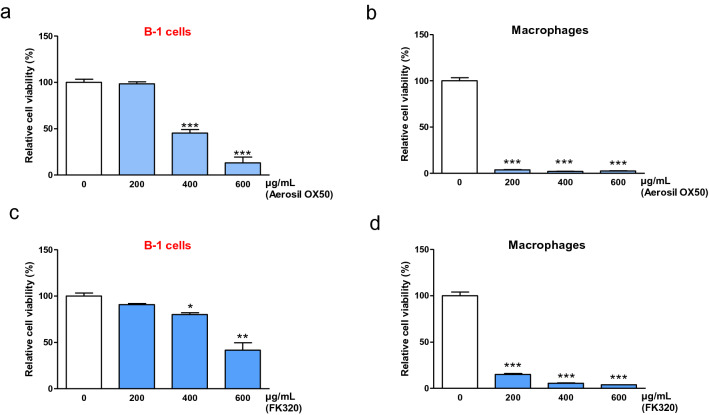
B-1 lymphocytes and macrophages respond to nanometric particles. **a**–**d** Relative cellular activity (ATP) measured in peritoneal B-1 lymphocytes (**a**, **c**) and macrophages (**b**, **d**) 24 h after exposure to different doses of nanometric silica particles (Aerosil OX50 **a**, **b** and FK320 **c**, **d**) compared to unexposed cells

**Fig. 7:**
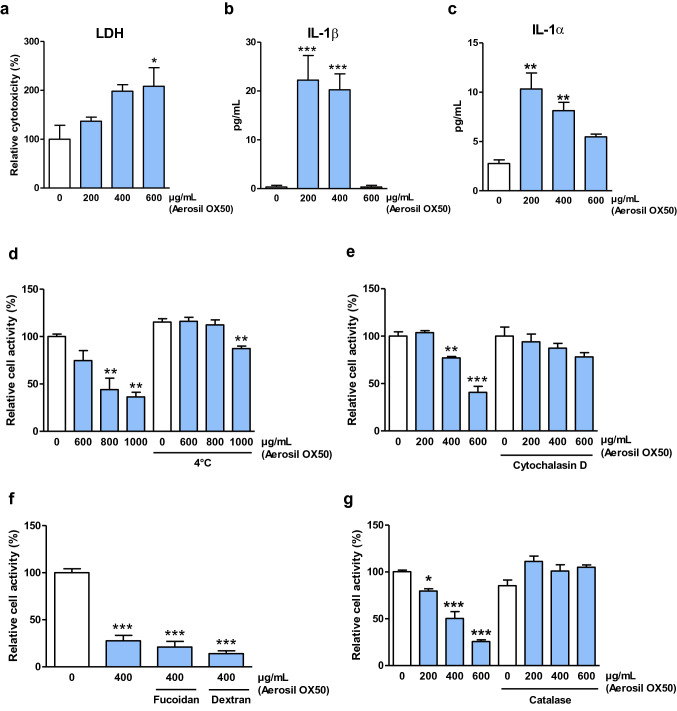
B-1 lymphocytes sense endocyte nanoparticles, die in an ROS-dependent manner and secrete proinflammatory cytokines. **a**–**c** Inflammatory mediator release by peritoneal B-1 lymphocytes after exposure to nanosilica (Aerosil OX50). Measured mediators are lactate dehydrogenase (LDH) (**a**), interleukin 1α (IL-1α) (**b**) and interleukin 1β (IL-1β) (**c**). Lipopolysaccharide (LPS, 0.1 μg/mL) was added as a positive control for IL-1β measurements. **d**–**g** Relative cellular activity (ATP) measured in cultures of peritoneal B-1 lymphocytes after nanosilica Aerosil OX50 exposure with different treatments to inhibit nanosilica cytotoxicity as 4 °C cell culture. (**d**) Cytochalasin D (2.5 μg/mL), (**e**) fucoidan (100 and 500 μg/mL) and dextran (100 μg/mL) (**f**) and catalase (**g**) compared to unexposed cells. Particle exposure lasts 4 h and not 24 h for the cold culture method

We next completed our in vitro study by determining whether B-1 lymphocytes respond to nanoparticles as macrophages. First, the survival of purified B-1 lymphocytes and macrophages exposed for 24 h to a range of nanosized silica doses was compared in vitro. In contrast to micrometric silica (Fig. 3a–d), nanosilica was cytotoxic to B-1 lymphocytes in a dose-dependent manner (Fig. 6a, c). Peritoneal macrophages were more affected than B-1 lymphocytes when compared at the same nanosilica doses (Fig. 6b, d). We then investigated whether nanosilica-exposed B-1 lymphocytes die from pyroptosis and membrane disruption as macrophages (Pavan et al. 2014, 2020). In contrast to micrometric crystalline silica (Figure S3e), we noted pyroptosis-associated IL-1β, alarmin-related IL-1α and LDH release in the supernatants of B-1 lymphocyte cultures exposed to nanosilica (Fig. 7a–c). These data indicated that B-1 lymphocytes are additional innate immune cells sensing nanoparticles and amplifying inflammatory responses caused by nanoparticles.

To determine by which mechanisms B-1 lymphocytes respond to nanosilica, specific inhibitors of particle-related recognition and pyroptosis were added to B-1 lymphocyte cultures exposed to cytotoxic doses of nanosilica. Similar to macrophages, B-1 lymphocytes internalize nanosilica since inhibiting endocytosis prevented nanosilica toxicity (Fig. 7d, e). We showed, however, that inhibition of scavenger receptors did not prevent toxicity in treated B-1 lymphocytes, indicating an unclassical mechanism of endocytosis (Fig. 7f) compared to crystalline silica (Nakayama 2018). Our data also showed that nanosilica toxicity is partly caused by reactive oxygen species production (ROS) because adding catalase reduced the toxic effects of nanosilica on B-1 lymphocytes (Fig. 7g). In conclusion, the sensing and endocytosis of nanoparticles by B-1 lymphocytes results in oxidative stress-induced cytotoxicity, pyroptosis and proinflammatory mediator release. B-1 lymphocytes thus participate in nanosilica-related inflammogenic responses as macrophages.

## Discussion

Inhalation of reactive particles leads to pulmonary granulomatous and fibrotic lesions and is associated with an increased risk of developing cancer and autoimmune diseases (Cullinan et al. 2017). The pathophysiological mechanisms of these diseases are incompletely understood, and lung disorders caused by particles remain highly refractory to existing therapies. The current paradigm for the detrimental effects of inhaled particles is mainly based on particle-activated macrophages. Indeed, the existing in vitro and in vivo models induced by particles rely on macrophages and their capacity to release pathogenic mediators (Kabadi et al. 2019; Sanchez et al. 2011). In this study, we identified an additional innate immune cell population, i.e., B-1 lymphocytes that respond to particles and accumulate in treated lungs during the time window during which granulomas and fibrosis are in development and when the switch from M1-polarized macrophages to more M2-polarized macrophages is operative (Xiang et al. 2016). In this study, we questioned the functions of recruited B-1 lymphocytes. Because they share many characteristics with macrophages, we first theorized that B-1 lymphocytes could participate in particle responses by ensuring similar or identical functions to macrophages. However, our data highlight new and distinct functions for B-1 lymphocytes during particle-induced granuloma formation.

B-1 lymphocytes in adults can arise from bone marrow precursors but predominantly proliferate from self-renewal resident cells located in the peritoneal cavity (Baumgarth 2017). It is likely that the accumulation of lung B-1 lymphocytes that we report originates from their peritoneal and pleural reservoirs. Indeed, using cell tracking, others have shown that B-1 lymphocytes are sensitive to an inflammatory signal and exit these reservoir cavities to migrate toward the inflammatory focus (Baumgarth 2011). In response to pathogens (influenza virus, *E. coli* and *S. pneumoniae*) B-1 lymphocytes are also mobilized toward the inflammatory site and draining lymph nodes (Aziz et al. 2015). Migration from the peritoneal cavity toward the lamina propria has also been reported after LPS and lipid A injection (Ha et al. 2006). Our present data resonate with the findings of others showing the recruitment of peritoneal and pleural B-1 lymphocytes into the lungs, lung-associated lymph nodes and spleen in asbestos-treated mice (Ferro et al. 2014; Pfau et al. 2014). Similar to our results, they showed that the migration of B-1 lymphocytes is dependent on fiber reactivity and lasts up to 8 months after the first exposure. Taken together, these data indicate that B-1 lymphocytes respond to inorganic particles and migrate from the peritoneal cavity to the inflammatory site and lymphoid organs.

Authors stressed the promiscuity of B-1 cells and M2 macrophages in the plurinuclear giant cells found in foreign-body granulomas. Bogsan et al. (2005) show that once peritoneal B-1 lymphocytes have migrated toward foreign bodies, some of them fuse with macrophages to form plurinuclear giant cells. This illustrates the proximity in their lineage, as B-1 lymphocytes are able to fuse with macrophages and combine their phagocyte functions to engulf particles, preventing both their dispersion in healthy tissues and excessive inflammation (Trout and Holian 2020b). Moreover, the deficiency in B-1 lymphocytes decreases the number of plurinuclear giant cells, while reconstitution with B-1 lymphocytes recovers their formation. This shows that the presence of B-1 lymphocytes is a determinant factor in the development of plurinuclear giant cells in foreign-body granulomas. Although the current paradigms of granuloma formation and particle responses are mainly based on macrophage activation, taken together, these data demonstrate that B-1 lymphocytes are also involved in granuloma development by promoting plurinuclear giant cell formation.

This research identified a novel characteristic of B-1 lymphocytes: their particle-clustering ability. This property seems specific to B-1 lymphocytes and reactive particles, as it was not observed using other cell types or particles. Taking into account that unlike macrophages, B-1 lymphocytes were resistant to the cytotoxicity of micrometric particles, we suggest that B-1 lymphocytes use particle clustering to group particles to avoid their distribution and act as a shield to protect surrounding cells from their cytotoxicity.

Using a deficiency/reconstitution model, Russo and Mariano (2010) showed that peritoneal B-1 lymphocytes arise in lung lesions upon exposure to the *bacterial Mycobacterium bovis*. These B-1 lymphocytes modify the cellular composition and organize the granuloma during the chronic phase. Recruited B-1 lymphocytes communicate with leukocytes to shape lesions into focal, compact granulomas, preventing the spreading of mycobacteria to other organs. Their research using in vivo experiments echoes our culture model and supports our observations on the clustering ability of B-1 lymphocytes.

In addition to clustering particles, we newly observed that B-1 lymphocytes participate in granuloma formation by shaping the particle-induced cellular aggregates of M2 macrophages into three-dimensional granuloma-like structures. Indeed, M2 macrophages form cellular aggregates when exposed to granulomagenic particles, and this effect was exacerbated by B-1 lymphocytes, as they led to larger organized aggregates. These responses are specific to B-1 lymphocytes, as they were not observed with B-2 lymphocytes. We found that B-1 lymphocytes stimulate M2 macrophage release of TIMP-1 during granuloma formation. The role of B-1 lymphocytes in extracellular matrix remodeling is consistent with the time window of their accumulation in the lungs, as it corresponds to the maturation of inflammatory granulomas to fibrotic granulomas. Unbalanced extracellular matrix remodeling by collagenase inhibitors is a key factor in fibrotic granuloma persistence in lung responses to reactive particles (Kabadi et al. 2019; Mohan et al. 2020).

The literature also supports a collaborative mechanism between B-1 lymphocytes and macrophages, indicating that while both cell types participate in granuloma formation, their functions during these responses differ in a complementary way. In our co-culture model, adding B-1 lymphocytes to macrophages increased the size of the cellular aggregates induced by granulomagenic particles, and the organization of the cell aggregates changed as they became granuloma-like structures. The collaboration that we observed between macrophages and B-1 lymphocytes with inorganic granulomagenic particles is also found with organic foreign body-induced granulomas in the literature. Vigna et al. (2006) carried out an in vitro model rather similar to ours to study granuloma formation caused by the fungus *P. brasiliensis* and observed only granulomas by associating B-1 lymphocytes and macrophages.

A potential limitation of our granuloma model is the origin of B lymphocytes used in this study. We collected naïve B-1 and B-2 lymphocytes from their main niches under homeostatic conditions (peritoneal B-1 lymphocytes and splenic B-2 lymphocytes). Since their localization could influence their responses, we cannot exclude that B cells coming from different organs or after particle exposure possess different functions or activities in our model. This concept should be investigated in futures studies.

Our results confirm that B-1 lymphocytes functionally differ from M2 macrophages during particle-induced fibrosis. B-1 lymphocytes harvested from the silicotic lung do not directly stimulate fibroblasts and are not immunosuppressive. Therefore, lung B-1 lymphocytes should not be misidentified as profibrotic Breg/B10 lymphocytes studied by others in response to silica (Chen et al. 2017; Liu et al. 2016; Lu et al. 2017). Their ability to indirectly promote fibrosis by exacerbating macrophage-derived fibroblast growth factor release remains unknown and should be investigated in future studies. In our study, we also determined whether B-1 lymphocytes possess early innate immune functions comparable to M1 macrophages implicated in inflammation and leukocyte recruitment. Supporting the idea that B-1 lymphocytes share functions with macrophages during the responses to particles, we found that B-1 lymphocytes also release proinflammatory mediators (IL-1α and IL-1β) when exposed to nanosilica (not microsilica). Although in a significantly lower amount than macrophages, these data suggest that as M1 macrophages, B-1 lymphocytes play a part in the inflammatory cocktail associated with nanoparticle responses, probably by undergoing pyroptosis and membranolysis (Pavan et al. 2020).

In light of our findings, we conclude that B-1 lymphocytes serve unique functions in response to toxic particle inhalation. In addition to responding specifically to granulomagenic particles, B-1 lymphocytes migrate to the exposed lungs where they do not phagocytose but cluster particles, form granulomas in conjunction with regulatory macrophages, and reorganize matrix protein organization. As inflammatory macrophages, innate-like B-1 lymphocytes sense endocytosis and participate in inflammatory responses to nanosilica.

## Supplementary Information

Below is the link to the electronic supplementary material.

Supplementary file1 (PDF 635 KB)
