# Supplementary material for: Mouse innate-like B-1 lymphocytes promote inhaled particle-induced in vitro granuloma formation and inflammation in conjunction with macrophages
Source: Arch Toxicol. 2021 Dec 21;96(2):585–99. doi: 10.1007/s00204-021-03200-2 (PMC8837577; doi:10.1007/s00204-021-03200-2)
Supplement: Supplementary file 1 — Supplementary file1 (PDF 635 KB) [file 204_2021_3200_MOESM1_ESM.pdf]

Mouse innate-like B-1 lymphocytes promote inhaled particle-induced *in vitro* granuloma formation and inflammation in conjunction with macrophages

Léa Hiéronimus, Raïssa Demazy, Laura Christiaens, Francine Uwambayinema, Jean-François Geuens, Youssof Yacoub, François Huaux.

Louvain centre for Toxicology and Applied Pharmacology (LTAP), Institut de Recherche Expérimentale et Clinique (IREC), Université catholique de Louvain (UCLouvain), Brussels, Belgium.

Correspondence: Léa Hiéronimus, Louvain centre for Toxicology and Applied Pharmacology (LTAP), Université catholique de Louvain, Avenue Hippocrate 57. bte B-1.57.06, 1200 Brussels, Belgium. Phone: 00-32-2-764.53.39 - Fax: 00-32-2-764.53.38 - email: lea.hieronimus@uclouvain.be

Fig. S1

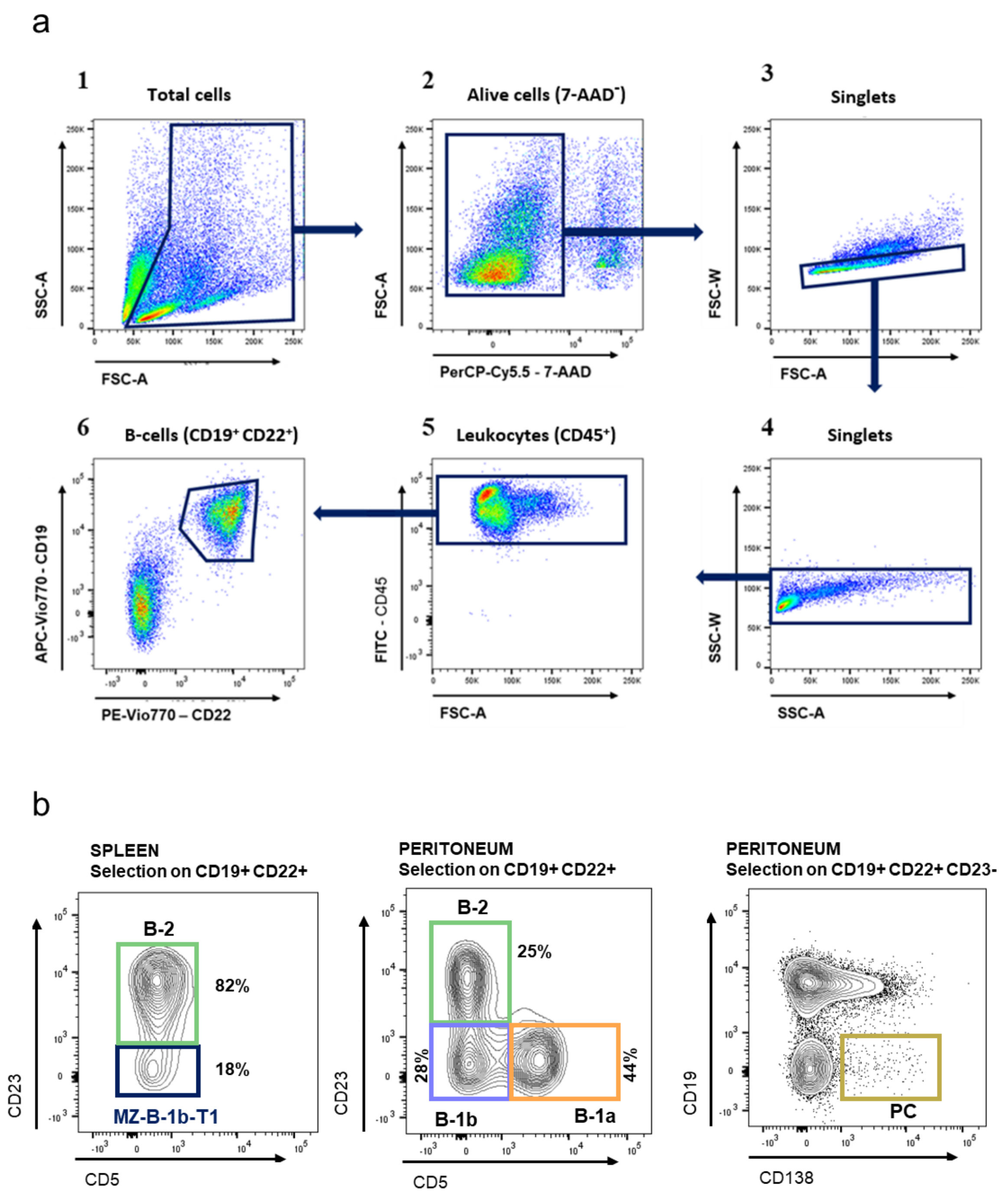

Supplemental figure 1 Gating strategy of B lymphocytes in the spleen (rich in B-2 lymphocytes) and in the peritoneum (rich in B-1 lymphocytes). **a** B-cell identification **1-6** Representative images of peritoneal cells. Debris exclusion and cell selection by FSC-A and SSC-A parameters (1). Alive (7-AAD$^-$) cell selection (2). Selection of singlet cells (3,4). Selection of leukocytes (CD45$^+$) cells (5). Selection of B lymphocytes (CD19$^+$CD22$^+$) (6). **b** Identification of B-1, B-2, MZ, transitional T1/T2 cells in the spleen (n=5) and of B-2, B-1a/b, PC cells in the peritoneum (n=3) of untreated mice

Fig. S2

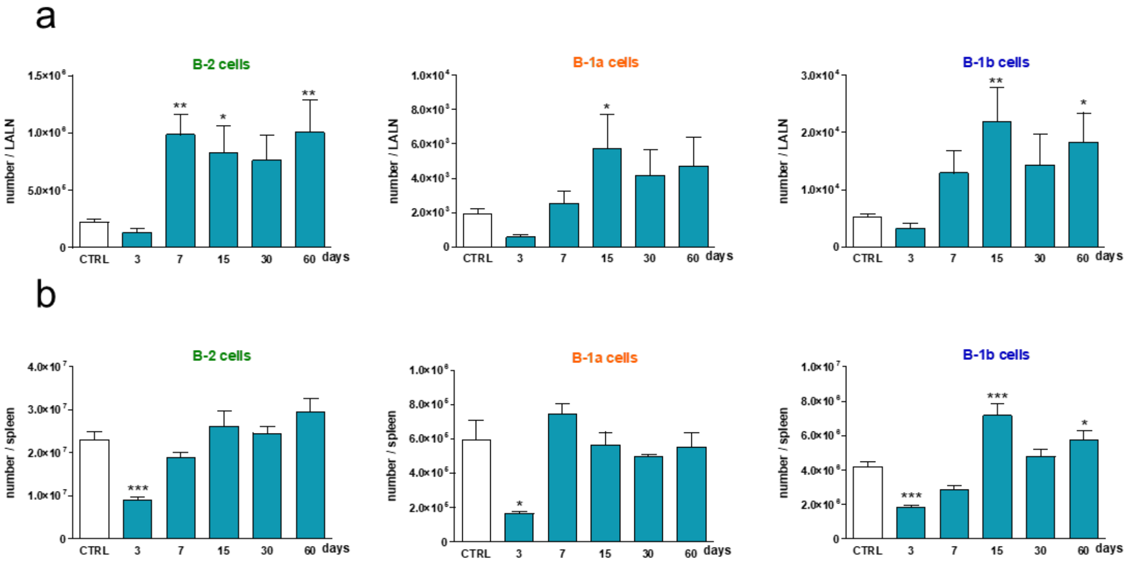

Supplemental figure 2 B-1 lymphocytes accumulated in the lung-associated lymph nodes of silica-treated mice but not in the spleen. **a, b** Number and percentage of B-1a, B-1b and B-2 lymphocytes in (**a**) the lung-associated lymph nodes and (**b**) the spleen of control mice or mice treated with silica (3, 7, 15, 30 and 60 days after treatment with SiO2, n=5 per time, 2.5 mg/mouse). The control column (CTRL) shows the combined results obtained from control mice analyzed at each studied time point (n=12, no significant difference between control groups, see material and methods)

Fig. S3

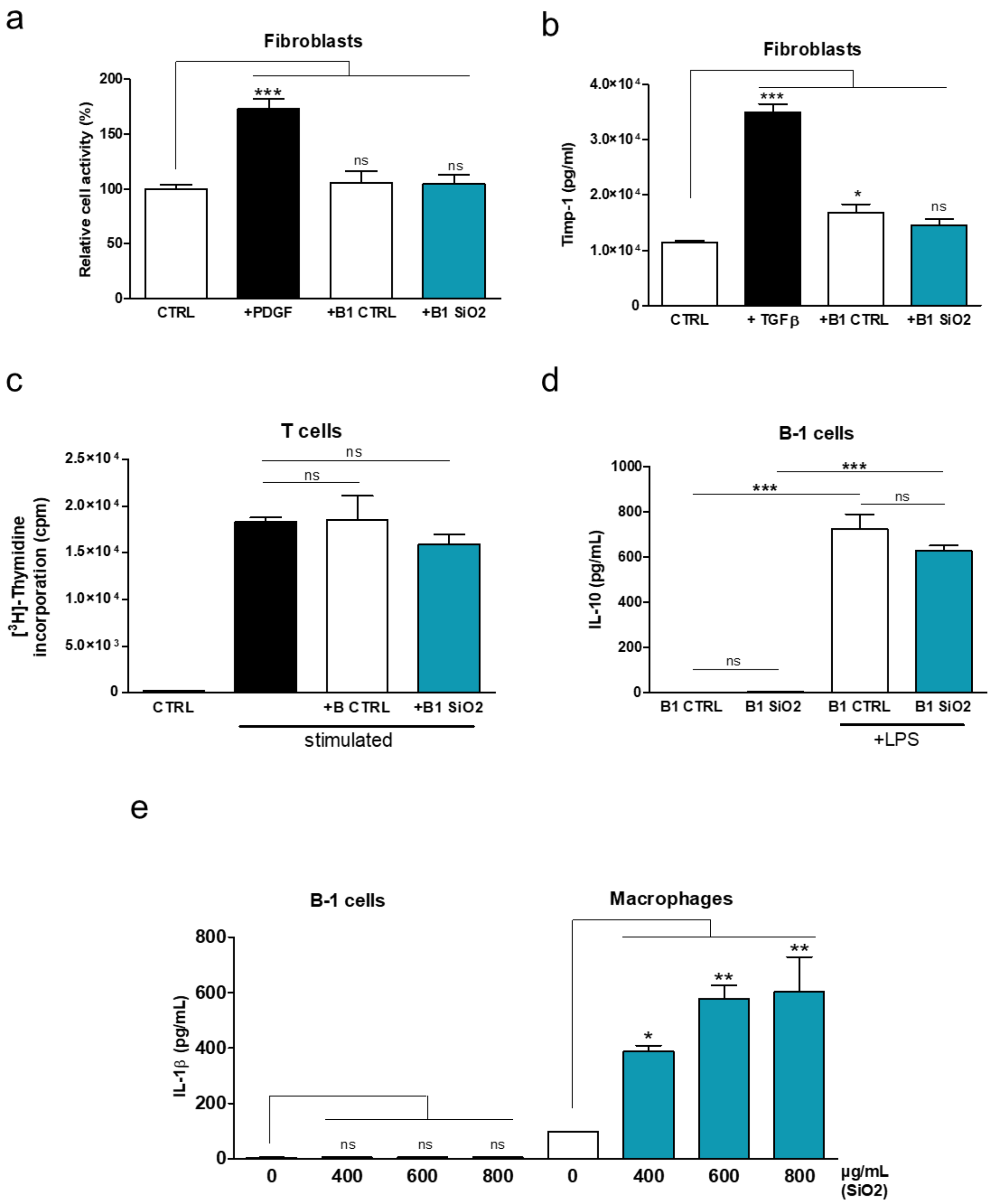

Supplemental figure 3 B-1 lymphocytes differ from inflammation- granuloma- and fibrosis-associated macrophages. **a, b** Quantification of (a) the cellular ATP content (relative cell activity) and (b) TIMP-1 levels in MLG fibroblasts (CTRL), stimulated (a: 30 ng/mL PDGF, b: 20 ng/mL TGF-β) or after coculture with B-1 lymphocytes purified from the lungs of control or silica-treated mice (15 days after treatment, 2.5 mg/mouse). **c** Inhibitory activity of B lymphocytes cocultured with activated $CD4^+$ T cells, measured by incorporation of $[^3H]$-thymidine. $CD4^+$ T cells were stimulated with anti-CD3 and anti-CD28 antibodies. B lymphocytes were purified from the lungs of control- or silica-treated mice (15 days after treatment). The ratio of the number of T-cells to the number of B-cells per well was 1:3. **d** Measure of IL-10 levels in culture supernatants of B-1 lymphocytes from control- or

silica-treated lungs after 48 hours of culture with or without LPS stimulation (1 µg/ml). **e** Measure of interleukin-1β (IL-1β) release by peritoneal B-1 lymphocytes or macrophages exposed to different doses of micrometric silica (DQ12) for 24 h. a-d: Bars represent the ± SD means of triplicates made from a pool of cells from 8 to 10 SiO2- or NaCl (CTRL)-treated mice. The statistical analysis graphed in c,d are Student's t-test. T lymphocyte proliferation, TIMP-1 release and cellular ATP content of splenic T cells or fibroblasts in coculture with B-1 lymphocytes purified from the lungs of NaCl- or silica-instilled mice were assessed based on the protocol described previously (Lebrun et al. 2017)
